# Role of Pigeons in the Transmission of Avian Avulavirus (Newcastle Disease-Genotype VIId) to Chickens

**DOI:** 10.3390/ani9060338

**Published:** 2019-06-10

**Authors:** Hany F. Ellakany, Ahmed R. Elbestawy, Hatem S. Abd El-Hamid, Rasha E. Zedan, Ahmed R. Gado, Ayman E. Taha, Mohamed A. Soliman, Mohamed E. Abd El-Hack, Ayman A. Swelum, Islam M. Saadeldin, Hani Ba-Awadh, Elsayed O.S. Hussein

**Affiliations:** 1 Poultry and Fish Diseases Department, Faculty of Veterinary Medicine, Damanhour University, El-Beheira 22511, Egypt; ellakany_hany@vetmed.dmu.edu.eg (H.F.E.); ahmed.elbestawy@vetmed.dmu.edu.eg (A.R.E.); drhatem_deltavet@yahoo.co.uk (H.S.A.E.-H.); rashazedan1987@gmail.com (R.E.Z.); ahmed.gado@vetmed.dmu.edu.eg (A.R.G.); 2 Department of Animal Husbandry and Animal Wealth Development, Faculty of Veterinary Medicine, Alexandria University, El-Beheira, Rasheed, Edfina 22758, Egypt; Ayman.Taha@alexu.edu.eg; 3 Poultry Diseases Department, Faculty of Veterinary Medicine, Minia University, El-Minia 61519, Egypt; mohamed.soliman@gmail.com; 4 Department of Poultry, Faculty of Agriculture, Zagazig University, Zagazig 44511, Egypt; dr.mohamed.e.abdalhaq@gmail.com or m.ezzat@zu.edu.eg; 5 Department of Animal Production, College of Food and Agriculture Sciences, King Saud University, P.O. Box 2460, Riyadh 11451, Saudi Arabia; aswelum@ksu.edu.sa (A.A.S.); isaadeldin@ksu.edu.sa (I.M.S.); vet.hani77@gmail.com (H.B.-A.); 6 Department of Theriogenology, Faculty of Veterinary Medicine, Zagazig University, Zagazig 44511, Egypt; 7 Department of physiology, Faculty of Veterinary Medicine, Zagazig University, Zagazig 44511, Egypt

**Keywords:** NDV genotype VIId, broilers, pigeons, contact infection, oropharyngeal, cloacal, shedding

## Abstract

**Simple Summary:**

Newcastle disease is an acute fatal disease of poultry. All broiler chickens and 8/15 pigeons were killed when infected intramuscularly (IM), while 7/10 chickens and only 1/15 pigeons were killed when infected intranasally (IN) with the virus in an experimental setting. Chickens in contact with infected pigeons developed severe respiratory, digestive and nervous signs. The mortality rates in chickens in contact with IM and IN infected pigeons were 2/5 and 3/5, respectively. Chickens in contact with IM infected pigeons had higher viral shedding titres than those in contact with IN infected pigeons. Free-range pigeons are considered an efficient carrier and transmitter of NDV-VIId to commercial broiler chickens raised in open houses.

**Abstract:**

Newcastle disease is an acute fatal disease of poultry. The aim of this study was to determine the dynamics of the transmission of avian avulavirus (velogenic viscerotropic Newcastle disease-genotype VIId) from either intramuscularly (IM)- or intranasally (IN) infected 8-week-old Egyptian Baladi pigeons in contact with commercial Arbor Acres broiler chickens (4 weeks of age). The mortality of IM infected chickens and pigeons was 10/10 for chickens and 8/15 for pigeons, while the mortality of IN infected chickens and pigeons was 7/10 for chickens and only 1/15 for pigeons. The concentration of viral shedding in the oropharynx was higher than that in the cloaca for both IN and IM infected pigeons. Pigeons infected IN continued shedding the virus from the oropharynx from the 4th day post-infection (dpi) up to the 16th dpi, while IM infected pigeons stopped oropharyngeal shedding at the 11th dpi. Chickens in contact with infected pigeons developed severe respiratory, digestive and nervous signs. The mortality rates in chickens in contact with IM and IN infected pigeons were 2/5 and 3/5, respectively. Chickens in contact with IM infected pigeons showed higher viral shedding titres in both the oropharynx and cloaca than chickens in contact with pigeons infected IN. In conclusion, free-range pigeons are considered an efficient carrier and transmitter of NDV-VIId compared to commercial broiler chickens raised in open houses.

## 1. Introduction

Avian avulavirus 1 or avian paramyxovirus type 1 is the cause of virulent Newcastle disease outbreaks characterized by high morbidity and mortality rates (up to 100%) in susceptible birds [1,2,3]. Newcastle disease is classified as a list A disease by the Office International des Epizooties [3]. Newcastle disease virus (NDV) class II contains at least 18 genotypes that include virulent and avirulent viruses [4,5,6,7]. Before 2013, all of the isolated NDV strains in Egypt belonged to class II, genotypes II and VI. Radwan et al. [8] first reported the incidence of three outbreaks of NDV class II, genotype VII, subgenotype d (lineage 5 sub-lineage d) in 2011–2012 in three broiler chicken flocks. One year later, Abdel-Glil et al. [9] and Hussein et al. [10] indicated that most of the recent circulating Egyptian NDV isolates from chickens belonged to class II, genotype VII, subgenotype d.

Birds in the Columbiformes order, which includes pigeons and doves, can be infected with NDV; however, the disease in pigeons is mostly caused by pigeon-specific viruses, which are known as pigeon paramyxovirus-1 (PPMV-1) genotype VIb to distinguish them from other avian paramyxoviruses type 1 viruses [11,12]. In contrast to the majority of wild birds, those Columbiform birds have been implicated as amplification or reservoir hosts that appear to be frequently infected with virulent strains of NDV [6,11,13].

Chicken NDV occurs in pigeons as a result of virus dissemination from affected chicken flocks, and it reoccurs in poultry flocks following the spread of the virus from affected domesticated or feral pigeons [14]. The viruses isolated from pigeons were classified by several studies as different genotypes, such as genotypes II, VI, and VII in class II [15,16,17,18].

No previous studies have compared the susceptibility of chickens and pigeons to infection by the same VVNDV genotype VIId strain. Therefore, the current study aimed to study the risk of dissemination of chicken NDV genotype VIId from experimentally infected pigeons in contact with broiler chickens via examination of the clinical, pathological, and immunological consequences and viral shedding titres from both bird species.

## 2. Material and Methods

### 2.1. Birds and Design for Experimental Infection 

Chickens: 50 broiler chickens (Arbor Acres) were purchased from NASCO Egypt poultry company, Alexandria, and raised until experimental infection with NDV at 4 weeks of age.

Pigeons: 40 squabs (Egyptian Baladi breed of different colours supplied from a local producer in Berma, Gharbia governorate) were raised from 1 day of age until infection at 8 weeks of age. One-day-old pigeons were purchased 4 weeks before 1-day-old commercial broiler chicks were purchased. The experimental design was presented in Table 1.

Birds were floor-reared in six separate rooms with exhausts and used for the experiment (6 groups including contact-infected chickens, G3c with pigeons in G3p and contact-infected chickens G4c with pigeons in G4p). All precautions were provided to prevent cross-contamination among the different rooms, as each room had a different entrance and was maintained by a different attendant (labour). The exhaust from each of the different rooms was received in a closed drainage cycle.

All birds were non-vaccinated against NDV or any other diseases. The birds were tested regularly (at 1, 2 and 4 weeks for chickens and 1, 2, 4, 6 and 8 weeks for pigeons) to detect the haemagglutination-inhibition (HI) antibody titres of NDV and were negative for NDV (HI titres = 0) at the age of experimental infection. The birds were also negative for the NDV genome, as determined by real-time reverse transcription polymerase chain reaction (rRT-PCR) using oropharyngeal and cloacal swabs on the day of infection. Birds were fed fresh feed and water ad libitum throughout the experimental period. All experimental tests and procedures complied with the general guidelines and were approved by the Local Ethics Commission of the Animal Health and Welfare of Damanhour University with respect to the care of birds, and all efforts were made to minimize suffering. The Ethical Approval Code is DMU/VetMed-2018-/0029.

At the end of the experiment (16 dpi), live moribund birds were killed humanely by intraperitoneal injection of 10–20 mg sodium pentobarbital/1 kg body weight (Nembutal; Sanofi-Phylaxia, Budapest, Hungary). For biosecurity reasons, all killed or dead birds that had been examined were soaked in 10% formalin disinfectant for 2 h and then burned in an incinerator.

### 2.2. Challenge Virus

The NDV used for challenge in this study was isolated from a 28-day-old broiler chicken flock with a capacity of 10,000 birds and a total mortality of 60% in El-Behera Province, Egypt. The intracerebral pathogenicity index (ICPI) and mean death time (MDT) of the virus were 1.89 and 48 h, respectively. The virus was genotyped as (VVNDV genotype VIId) NDV/EG/CK/18/2015 with GenBank accession number (KU377781). The cleavage site amino acid of the F protein contains arginine (R) at positions 112, 113 and 116, lysine (K) at position 115 as R-R-Q-K-R, and a phenylalanine (F) at position 117 [17]. The egg infectious dose (EID_50_) was 10^6.3^/0.2 mL/chick and was administered either intramuscularly (IM) or intranasally (IN) [13,19,20]; the EID_50_ was calculated according to the method of Reed and Muench [21]. This challenge virus was prepared for analysis by inoculation into 10-day-old SPF eggs obtained from local SPF farms in Kom Osheim, El-Fayoum, Egypt.

### 2.3. Clinical Observation

All birds were observed daily for clinical signs, mortality rate, and post-mortem (PM) lesions for 16 dpi.

#### 2.3.1. HI Test

For HI testing, 10 blood samples from each chicken and pigeon group infected IN or IM and 5 blood samples from each chicken group infected by contact were collected at 0, 4, 7, 11 and 16 dpi.

A total of 0.025 mL of each serum sample was double-fold serially diluted in phosphate-buffered saline (PBS) across a plastic U-bottomed microliter plate. Four haemagglutinin units (HAU) of virus/antigen were added in the same quantity to each well and allowed to rest for a minimum of 30 min at room temperature (i.e., approximately 20 °C), and 0.050 mL of 0.5% (*v*/*v*) chicken RBCs was added to each well. After being gently mixed, RBCs were allowed to settle to a distinct button for approximately 40 min at room temperature. Positive and a negative control sera were applied in two rows. The HI titre was the highest dilution of serum causing complete inhibition of 4 HAU of antigen.

HI titres were regarded as positive if there was inhibition at a serum dilution of 1/16 (4 log_2_ when expressed as the reciprocal) or more against 4 HAU of antigen [3].

#### 2.3.2. Determination of Viral Shedding via rRT-PCR

Oropharyngeal and cloacal swabs were individually collected from five birds/group at 4, 7, 11 and 16 dpi and then pooled for RNA extraction for the detection of viral shedding. RNA was extracted using QIAamp Viral RNA Mini-kits (52904; Qiagen, Hilden, Germany), and rRT-PCR was performed (3005P; Strategen, La Jolla, CA, USA) with Quantitect RT-PCR Kit reaction buffer (204443; Qiagen) according to the manufacturer’s protocol. The following primers were used to identify NDV targeted fusion (F) proteins, according to Wise et al. [22]:

Velo- and mesogenic (F+4839): 5′-TCCGGAGGATACAAGGGTCT-3′; Fusion (F+4894-VFP-1): 5′-[FAM]AAGCGTTTCTGTCTCCTTCCTCCA[TAMRA]-3′; 101 bp (F-4939): 5′-AGCTGTTGCAACCCCAAG-3′. Primer preparation was set according to the manufacturer’s instructions to obtain a 100 pmol/µL primer. For each primer set, the RT step was 30 min at 50 °C, followed by 15 min at 95 °C. The cycling conditions for the NDV F gene primers consisted of 40 cycles of 10 s of denaturation at 94 °C, 30 s of annealing at 58 °C, and extension at 72 °C for 10 s.

#### 2.3.3. Statistical Analysis

Statistical measurements of mortality frequencies were analysed by the Chi-square test using Statistical Analysis System (SAS) [23], and data frequencies were considered significant at *p* < 0.0001; the data obtained from the HI assay were handled with the SPSS programming tool (IBM SPSS. 20^®^, Coppell, TX, USA) using one-way ANOVA, and *p* < 0.05 was considered statistically significant.

## 3. Results

Clinical Signs (Table 2), Mortality Rates, and PM Lesions.

### 3.1. Broiler Chickens Infected IM (G1c) and IN (G2c)

Both groups had pronounced respiratory, digestive, and nervous signs consistent with NDV, indicating the high pathogenicity of our NDV-VIId challenge strain in non-vaccinated chickens. Severe depression; respiratory signs, such as gasping, sneezing, conjunctivitis, head swelling, and ocular and nasal discharge; and watery greenish diarrhoea appeared in G1c and G2c on the 1st and 2nd dpi. Nervous signs began early with head shaking and tremors on the 3rd dpi in birds of G1c and on the 15th dpi in birds of G2c. The sick birds in G2c showed complete torticollis on the 16th dpi.

**Figure 1:**
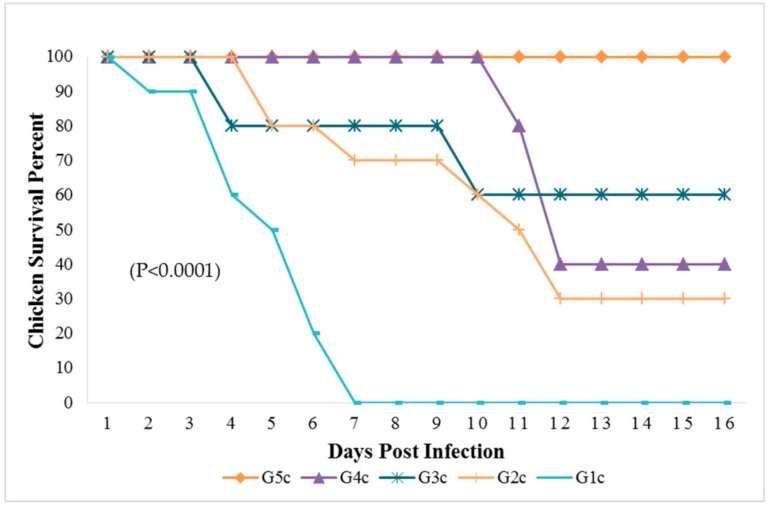
Survival percent in chicken groups until the 16th dpi analyzed by Chi-square test using statistical analysis system; SAS data frequencies were significant at (*p* < 0.0001). G1c: n = 10, G2c: n = 10, G3c: n = 5, G4c: n = 5, and G5c: n = 10.

**Figure 2:**
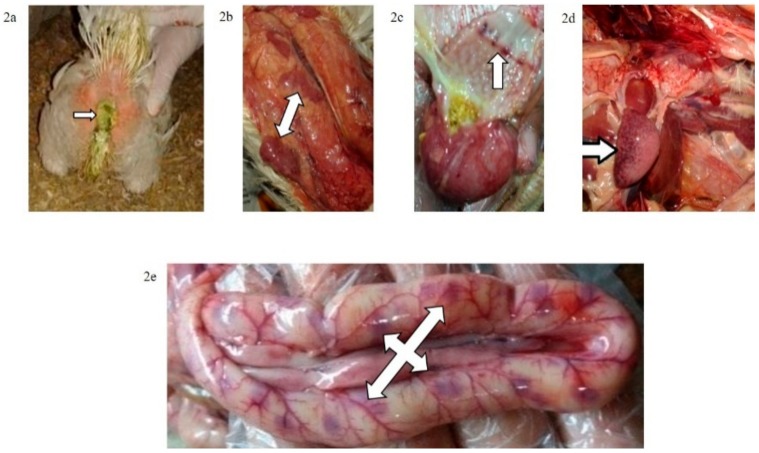
(**a**) Greenish diarrhoea in a broiler chickens infected IM with NDV-VIId (G1c); (**b**) congestion in the thymus of a broiler chicken in G1c; (**c**) petechial haemorrhage at the junction between the oesophagus and proventriculus of a broiler chicken in G1c; (**d**): button-like ulcer in the intestine of a broiler chicken in G1c; (**e**) enlarged mottled spleen of a broiler chicken in G1c.

**Figure 3:**
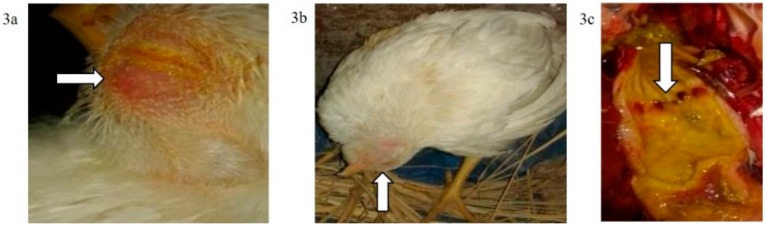
(**a**) Conjunctivitis and swelling of the head of a broiler chicken infected IN (G2c); (**b**) torticollis in a broiler chicken infected IN with NDV-VIId (G2c); (**c**): petechial haemorrhage at the junction between the oesophagus and proventriculus of a broiler chicken infected IN with NDV-VIId (G2c).

The mortality rate was 10/10 (100% during the first six dpi) in chickens of G1c (Figure 1) and 7/10 (70%) in those of G2c and these mortality rates were significantly higher (*p* < 0.0001) than those in the control group (G5c). The PM examination revealed severe tracheitis and petechial haemorrhages between the oesophagus and the proventriculus, button-like ulcers in the intestine, congestion of the thymus, enlarged and darkly congested spleens, and nephritis (swollen, spotted and pale kidneys) in both groups G1c and G2c (Figure 2a–e and Figure 3a–c, respectively).

### 3.2. Pigeons Infected IM (G3p) and IN (G4p)

**Figure 4:**
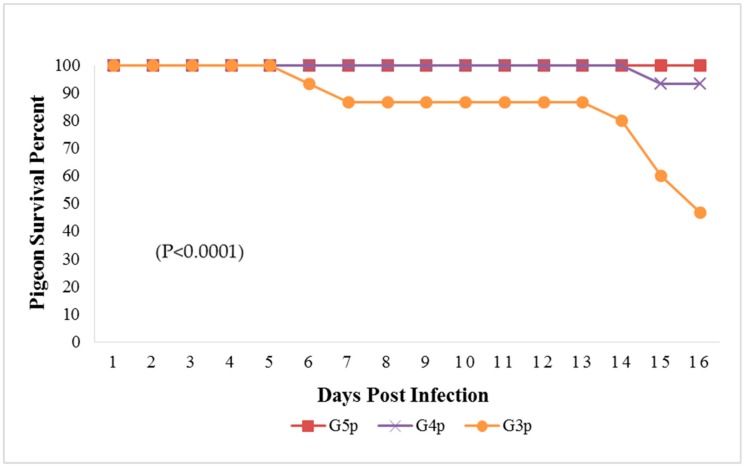
Survival percent in pigeon groups until the 16th dpi analyzed by Chi-square test using statistical analysis system SAS; data frequencies were significant at (*p* < 0.0001). G3p: n = 15, G4p: n = 15, and G5p: n = 10.

**Figure 5:**
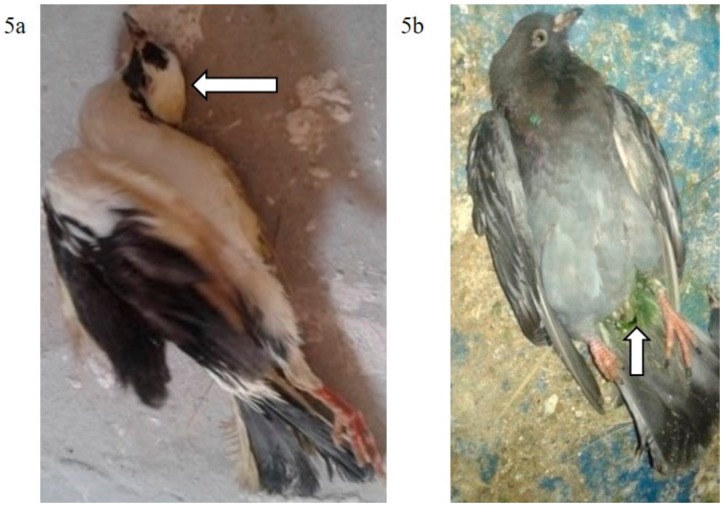
(**a**): Torticollis in a pigeon infected IM with NDV-VIId (G3p); (**b**): Greenish diarrhoea in a pigeon infected IN with NDV-VIId (G4p).

The mortality rate in G3p was 8/15 (53.33%), while in G4p, it was 1/15 (6.66%), which were significantly higher (*p* < 0.0001) than that of the control group (G5p) until the end of the experiment (Figure 4). IM infected pigeons, G3p, showed depression on the 3rd dpi, and mild respiratory signs such as conjunctivitis, swollen head and gasping were observed on the 3rd, 5th and 8th dpi, respectively. Greenish diarrhoea started on the 6th dpi. The apparent nervous signs of pigeons included head tremors, torticollis and leg paralysis and appeared later (13th dpi) in G3p (Figure 5a) than in G4p (pigeons infected IN). IN infected pigeons, G4p, showed depression, respiratory signs and greenish diarrhoea (Figure 5b), but these signs were delayed in pigeons to the 8th dpi, and all clinical signs were less severe in this group than in G3p. The PM lesions observed in pigeons of both G3p and G4p were tracheitis, liver congestion, nephritis, and congestion of brain blood vessels. The lesions of the birds in G4p were less severe than those in G3p.

### 3.3. Chickens in Contact with Pigeons Infected IM (G3c) and IN (G4c)

**Figure 6:**
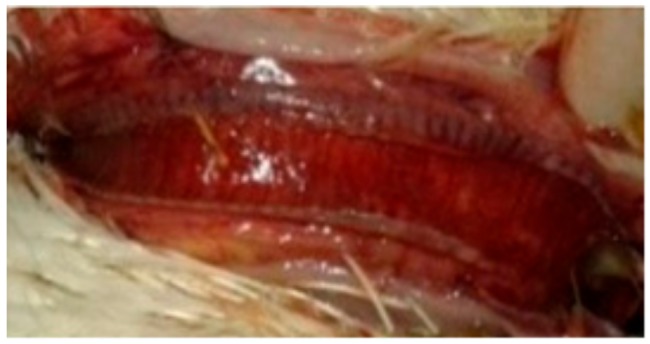
Congested trachea of a broiler chicken in contact with pigeons infected IN with NDV-VIId (G4c).

The broiler chickens in contact with pigeons showed depression, conjunctivitis and head swelling on the 3rd dpi in G3c and on the 8th dpi in G4c, whereas gasping was observed on the 6th dpi in G3c and on the 9th dpi in G4c. Nervous signs in the form of head tremors and leg paralysis were delayed to the 13th dpi. The PM lesions consisting of tracheitis were more severe in G3c than in G4c (Figure 6), while the haemorrhages at the junction between the oesophagus and the proventriculus were observed on the 4th dpi. Mortality occurred on the 4th and the 10th dpi in 2/5 (40%) of the birds in G3c, but in G4c, mortality occurred later at the 11th and 12th dpi in 3/5 (60%) of the chickens. These mortality rates were significantly higher (*p* < 0.0001) than those in the control group (G5c) (Figure 1).

None of the pigeons or chickens in the non-infected controls G5p and G5c showed any signs of disease or death throughout the experiment.

### 3.4. NDV HI Test 

**Figure 7:**
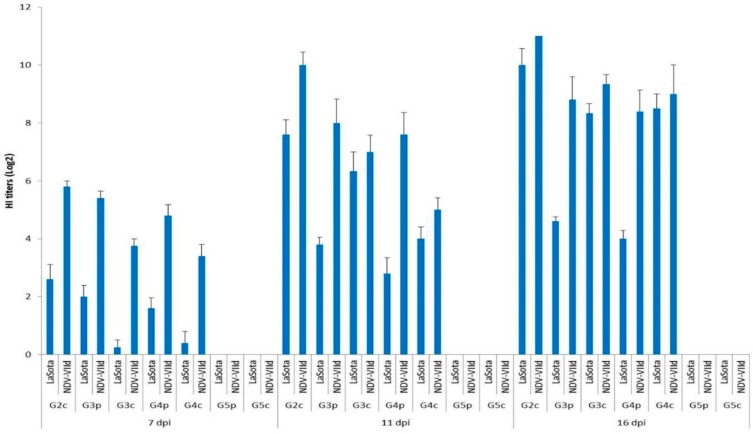
Mean HI titres (log2) for all bird groups at 7, 11, and 16 dpi using LaSota and NDV-VIId antigens. G1c: n = 10, G2c: n = 10, G3p: n = 15, G3c: n = 5, G4p: n = 15, G4c: n = 5, G5p: n = 10, and G5c: n = 10.

All birds (pigeons and broiler chickens) were negative for HI antibodies on the same day as infection (0 dpi). Nonspecific HI titres (<1) appeared on the 4th dpi. All chickens infected IM in G1c were dead before the end of the 7th dpi; thus, no HI testing could be performed for this group. The HI titres of broiler chickens infected IN (G2c) using the homologous NDV-VIId antigen were significantly higher (*p* < 0.05) than those titres estimated by the LaSota antigen on the 7th and on the 11th dpi, with values of 5.80 vs. 2.60 and 10.00 vs. 7.60 log_2_, respectively. The titres with the NDV-VIId antigen were high (non-significantly, *p* > 0.05) on the 16th dpi, with 11.00 vs. 10 log_2_, respectively, with both antigens (Figure 7).

Statistical analysis was applied using the SPSS programming tool (IBM SPSS. 20^®^, Coppell, TX, USA) using one-way ANOVA, and *p* < 0.05 was considered statistically significant. G1c = HI was not applied because all chickens infected IM were already dead by the 7th dpi.

The titres of the pigeons infected IM (G3p) and those of pigeons infected IN (G4p) were compared, and it was determined that the titres measured by the NDV-VIId antigen were higher than those measured by the LaSota antigen on the 7th, 11th, and 16th dpi, with values of 5.40, 8.00 and 8.80 log_2_ vs. 4.80, 7.60 and 8.40 log_2_, respectively.

Then, the HI titres of infected pigeons were compared with those of the chickens with which they had contact, G3p and G3c, by measuring the homologous NDV-VIId antigen, and the HI titres were higher in pigeons than in their contact-infected chickens on the 7th and on the 11th dpi, with values of 5.40 vs. 3.75 and 8.00 vs. 7.00 log_2,_ respectively. However, on the 16th dpi, the situation had an opposing trend, and the values were reversed, as the contact-infected chickens showed higher titres than their pigeon mates (9.33 vs. 8.80 log_2_, respectively). On the other hand, when using the heterologous LaSota antigen, the values of pigeons’ serum HI titres were higher than those in their contact-infected chickens only on the 7th dpi (2.0 vs. 0.25 log_2_). However, the situation was also reversed on the 11th and 16th dpi when the contact-infected chicken HI titres were higher than those of their pigeon mates (6.33 vs. 3.80 log_2_ and 8.33 vs. 4.60 log_2_, respectively). In the same context, pigeons infected IN, G4p, were compared with their contact-infected chickens, G4c, and the HI titres measured by the homologous NDV-VIId antigen were higher in pigeons than in their contact-infected chickens on the 7th and on the 11th dpi (4.80 vs. 3.40 and 7.60 vs. 5.00 log_2_, respectively). Meanwhile, the titres on the 16th dpi did not follow the same pattern, as the HI titres were higher in contact-infected chicken than in their infected pigeon mates. The titres were higher in the infected pigeons than in their contact-infected chickens only on the 7th dpi when the LaSota antigen was used, with values of 1.60 vs. 0.40 log_2_, respectively. Meanwhile, on the 11th and 16th dpi, the HI titres of the contact-infected chickens were higher than those of their infected pigeon mates (4 vs. 2.80 and 8.50 vs. 4 log_2_, respectively).

All chickens (G5c) and pigeons (G5p) of the control groups showed (0.00 log_2_) HI antibody titres throughout the experiment, as measured by both antigens.

### 3.5. NDV Genotype VIId Shedding after Experimental Infection

Oropharyngeal and cloacal swabs from chickens of G1c (infected IM) that died on the 6th dpi contained 6.30 and 5.96 log_10_ viruses, respectively. Chickens infected IN (G2c) had no oropharyngeal viral shedding either on the 11th or on the 16th dpi.

After the pigeons in G3p were IM infected, they excreted the virus from the 4th to the 7th dpi in the oropharynx with no oropharyngeal viral shedding on the 11th or on the 16th dpi, while the same pigeons excreted the virus in the cloaca from the 4th to the 11th dpi. Pigeons in G4p (infected IN) had no cloacal shedding on the 4th, 7th, or 11th dpi.

The quantity of oropharyngeal viral shedding in pigeons infected IN (G4p) was lower than that in pigeons infected IM (G3p), and viral shedding occurred between the 4th and 16th dpi. Cloacal shedding appeared only later at the 16th dpi in pigeons in G4p (infected IN).

**Figure 8:**
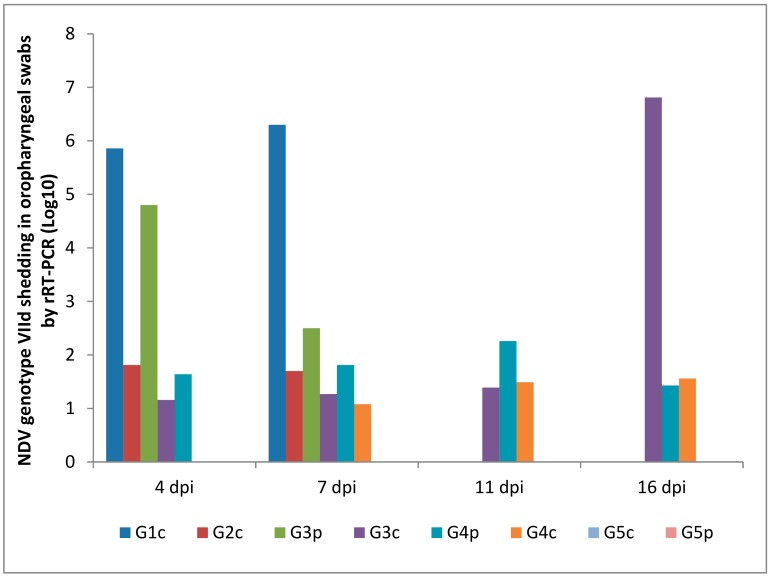
NDV genotype VIId oropharyngeal shedding by rRT-PCR (log _10_). G1c: n = 10, G2c: n = 10, G3p: n = 15, G3c: n = 5, G4p: n = 15, G4c: n = 5, G5p: n = 10, and G5c: n = 10.

**Figure 9:**
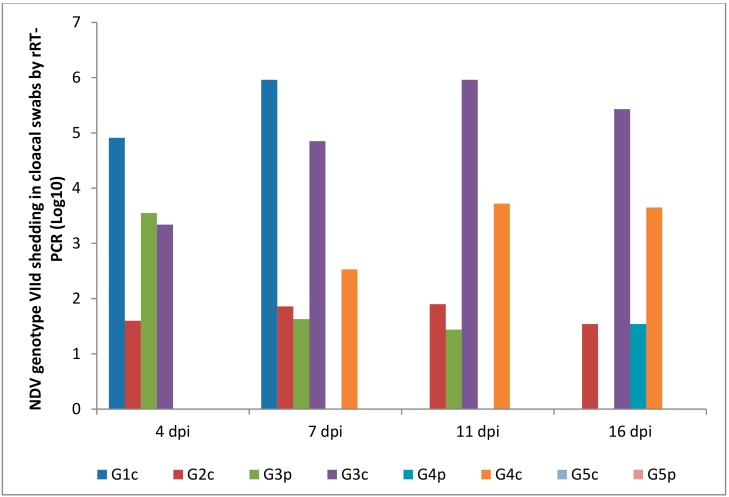
NDV genotype VIId oropharyngeal shedding by rRT-PCR (log _10_). G1c: n = 10, G2c: n = 10, G3p: n = 15, G3c: n = 5, G4p: n = 15, G4c: n = 5, G5p: n = 10, and G5c: n = 10.

Chickens in G3c (in contact with pigeons infected IM) showed viral shedding that peaked on the 16th dpi (6.81 and 5.43 log_10_) in the oropharynx and cloaca, respectively. However, chickens in contact with pigeons infected IN (G4c) were negative on the 4th dpi and then peaked on the 16th dpi (1.56 and 3.65 log_10_) in the oropharynx and cloaca, respectively (Figure 8 and Figure 9).

## 4. Discussion

Worldwide, NDV isolates from pigeons are placed into class II, genotypes II, VI and VII [15,16,17,24]. Understanding how these highly virulent viruses are maintained in pigeons is important for clarifying the risk factors that substantially contribute to the introduction of this virus to domestic poultry. Therefore, this study aimed to investigate the dynamics and behaviour (multiplication, seroconversion, lesions and mortality pattern, excretion in oropharynx or cloaca) of a recent NDV-VIId isolate in 8-week-old pigeons and contact-infected 4-week-old broiler chickens.

Birds of the Columbidae family (pigeons and doves) have been implicated as reservoir species for virulent strains of NDV in North America [25], while PPMV-1 is a class II, genotype VIb avian avulavirus and is host-adapted to pigeons and other Columbiform birds. These viruses are virulent variants of avian avulavirus that have circulated in and adapted to pigeons [26].

The severe nervous signs and paralysis in the experimentally-infected pigeons in this study were similar to those reported by Marlier and Vindevogel [27], who indicated that the clinical signs observed in pigeons were similar to those induced by neurotropic NDV (tremor of the neck and wings, torticollis, paralysis, and ataxia). In this study, the nervous signs and greenish diarrhoea started 3 days earlier in pigeons infected IM than in those infected IN. Guo et al. [28] also noticed only mild respiratory signs in pigeons naturally infected with virulent NDV. In our experiment, the respiratory signs started in pigeons infected IM on the 3rd dpi, whereas those infected IN showed signs on the 6th dpi in the form of gasping, head swelling, and conjunctivitis.

It is worth mentioning that the virus was administered IM herein only to examine the maximum susceptibility of pigeons to infection expressed as a mortality rate and to provide a standard for comparison of susceptibility with IN infected pigeons, which was very close to the natural route. Pigeons infected IM suffered from a high mortality rate of 8/15 (53.33%), and those inoculated IN showed a lower mortality rate of 1/15 (6.66%), which is consistent with many previous studies. Erickson et al. [29] reported that eye-drop instillation of a velogenic viscerotropic strain caused observable clinical disease (mild tremors) in only 1 out of 10 inoculated birds. Furthermore, Cattoli et al. [30] inoculated adult pigeons via eye drops with VVNDV, and clinical signs of head tremors, wry neck, opisthotonos, wing droop and leg paralysis were observed in only 5 out of 10 adult birds, and only 1 in 10 died. In addition, Alexander and Parsons [14] reported that pigeons inoculated IN with PPMV-1 did not show mortality. Higher mortality rates in pigeons were shown from field data than in experimental settings. Marlier and Vindevogel [27] indicated that the mortality rate of pigeons in contact with classical velogenic NDV-infected chickens was usually 40%, while the mortality rate in infected chickens reached 100% with the same virus. However, when pigeons were infected with PPMV-1, the mortality was much milder (10%).

It is possible that the difference in the mortality rate between the different studies is due to age differences. Cattoli et al. [30] recorded mortality in 7/21 juvenile squabs, whereas only 1 adult died out of 10. Alexander et al. [31] concluded that clinical signs in pigeons vary mainly according to age. In young birds, mortality can reach up to 100%, whereas in adults, morbidity is approximately 10%, and mortality is minimal.

Herein, pigeons infected IN showed clinical signs and lesions that were less severe than those in pigeons infected IM, with a moderate degree of tracheitis, liver congestion, nephritis, and congestion of brain blood vessels occurring in both groups on the 14th dpi. Wakamatsu et al. [32] described that grossly, pigeons infected with viscerotropic velogenic NDV showed only moderate spleen enlargement, as well as histological lesions of perivascular cuffing and gliosis in the cerebellum and brain stem by the 14th dpi. Pearson et al. [20] added that gross lesions in pigeons infected with PPMV-1 from natural outbreaks consisted of pancreatic necrosis, enteritis, and proventricular haemorrhages. Vindevogel and Duchatel [33] indicated that the most prominent clinical signs in young pigeons were mainly nervous signs and diarrhoea.

The mortality rate in chickens in contact with pigeons infected IN was higher than in those in contact with pigeons infected IM (60% vs. 40%), which is in contrast to the expected result that the rate of mortality in the chickens in contact with IM infected pigeons might be higher. This can be explained by the higher death rate (8/15) in pigeons infected IM within a short period, whereas in IN infected pigeons, the survival rate was 14/15, which provided an opportunity for longer exposure to a larger number of living pigeons shedding the virus.

Lesions associated with NDV in pigeons vary according to the virulence and source of the strains. In general, isolates from chickens, even when highly virulent, cause minimal pathological changes in pigeons [34], whereas the pigeon variant of NDV (PPMV-1) can cause a series of lesions that vary based on age and route of inoculation [32].

The HI test was performed in this study to investigate three questions: First, whether the source of the HI test antigen (homologous or heterologous) used in the test influenced the results; second, whether the route of infection among the birds of the same species influenced the results; and third, whether there was a difference between the infected pigeons and the chickens with which they had contact regarding the seroconversion to NDV.

From the abovementioned results, the titres measured with the homologous NDV-VIId antigen produced higher values (significantly at *p* < 0.05 in most cases) than those measured using the heterologous LaSota antigen, with a maximum difference of 4.4 log2. Significant differences were observed in the first measurement on the 7th dpi, but at later times (on the 11th and on the 16th dpi), the differences were lower than those in the first testing. This was similar to the results of Miller et al. [35] and Sedeik et al. [36], who found that HI antibody titres vary depending on the type of antigen used, either homologous or heterologous, depending upon the genetic dissimilarity between the LaSota and NDV-VIId antigens in the haemagglutinin-neuraminidase (HN) protein [37]. However, our results contradicted those of Perozo et al. [38], who reported that the genotype difference does not reflect the antigenic arrangements or serology but only reflects the NDV behaviour.

The comparison of the two avian species, pigeons and chickens, using the HI test, indicated that the infected chickens consistently had higher HI titres than pigeons at all testing periods regardless of the type of HI antigen used. Our data also indicated that IM infected species consistently produced higher HI titres than IN infected species, regardless of the species infected. Furthermore, we emphasize that on the 7th and the 11th dpi, contact-infected chickens showed lower HI titres than their infected pigeon mates, whereas at the 16th dpi, the contact-infected chickens showed higher HI titres than the pigeons. These data emphasize the importance of thoroughly studying the virus–host relationship because each species shows a specific pathobiology pattern important in the epidemiology of NDV [39].

The route of infection affected the start and the end of shedding and the concentration of shed viruses. In IM infected pigeons, virus shedding stopped after the 7th dpi in the oropharynx and after the 11th dpi in the cloaca. In IN infected pigeons, oropharyngeal shedding continued to the 16th dpi in both the oropharynx and cloaca. This indicated the higher and faster start of replication of the virus when IM was administered than when IN administered, and the IN administered virus led to slow progress in multiplication and shedding, which lasted for a longer time until the end of the experiment. Alexander et al. [31] found that the viral shedding observed begins on the 2^nd^ dpi. Furthermore, in the literature, pigeons experimentally infected with a highly pathogenic strain of chicken NDV administered via eye drops or IN shed the virus through both the cloaca and mouth from the 5th to the 24th dpi [29,39,40]. IM infected pigeons had a higher mortality rate of 8/15 during the first 10 dpi, and they started to shed the virus from the cloaca earlier than IN infected pigeons, which started shedding the virus later and had a high rate of survival (14/15).

Alexander and Parsons [14] and Toro et al. [41] added that feed contaminated with faecal matter of feral pigeons infected with NDV may act as a source of NDV infection in chicken flocks. Liu et al. [15] concluded that biosecurity measures should be strengthened to prevent the cross-infection of NDV between pigeons and chickens. PPMV-1 most likely originated after multiple events of interspecies transmission, which illustrates the role of pigeons in modifying the genetic makeup of chicken NDV, and chicken-pigeon transmission must have taken place much earlier than the early 1980s. Furthermore, a significant adaptation and evolution of the pigeon-type NDV variants has already occurred [42].

Carrasco et al. [39]; Vindevogel and Duchatel [43]; Carrasco et al. [44]; and Dortmans et al. [45] reported that both chickens and pigeons infected with either the virulent or non-virulent PPMV-1 strain excreted the virus at levels comparable to each other. Alexander and Parsons [14] also noticed that when PPMV-1 was IN inoculated into pigeons, seroconversion and viral excretion were observed for up to 31 dpi.

Viral shedding in the oropharynx on the 16th dpi from chickens in contact with pigeons infected IM was 100,000 times higher than that of chickens in contact with IN infected pigeons (6.81 vs. 1.56 log_10_). Likewise, viral shedding in the cloaca of chickens in contact with pigeons infected IM was approximately 100 times higher than that in the chickens in contact with IN infected pigeons (5.43 vs. 3.65 log_10_). However, we should not judge the effect of the route of infection of pigeons on the basis of the longevity and concentration of viruses shed from the chickens with which they had contact strictly because in both cases (IM or IN administered), pigeons and their contact-infected chickens were in the same enclosure for the whole period of the experiment (16 days), and they were sharing the virus at all times and in both directions, first catching the virus and then shedding it.

Nevertheless, it is worth mentioning that the concentrations of viral shedding in the experimental model are much higher than those under the field situation. However, chickens infected IN suffered from 70% mortality and stopped shedding the virus in the oropharynx on the 7th dpi and in the cloaca after the 16th dpi.

## 5. Conclusions

Regardless of the route of infection in pigeons, the concentration of viral shedding was higher in the oropharynx than in the cloaca. Chickens in contact with infected pigeons not only developed severe clinical signs and mortality but also showed higher and longer viral shedding in the cloaca than in the oropharynx. Contact-infected chickens showed a continuous rise in their serum HI titres until the 16th dpi. It is recommended to use homologous antigens in the HI test because using non-homologous antigens leads to misleading, inaccurate, and lower titres.

More comprehensive studies regarding the increased susceptibility of pigeons to velogenic NDV should be carried out. Vaccination of pigeons with live and inactivated NDV vaccines is recommended to control disease infection in pigeons. Free-range or loft pigeons are considered an efficient carrier and transmitter of NDV-VIId to commercial chicken flocks raised in an open housing system.

## Figures and Tables

**Table 1:** Experimental design.

Group	Type and No. of Birds	Dose of Infection	Age of Infection
G1c	10 broiler chickens infected IM	10^6.3^ EID_50_/0.2 mL/bird	4 weeks
G2c	10 broiler chickens infected IN	10^6.3^ EID_50_/0.2 mL/bird	4 weeks
G3p	15 pigeons infected IM and in contact with 5 G3c chickens	10^6.3^ EID_50_/0.2 mL/bird	8 weeks
G3c	5 broiler chickens in contact with G3p (placed in contact with infected pigeons 24 h after being IM infected)	Contact infection	
G4p	15 pigeons infected IN and in contact with 5 G4c chickens	10^6.3^ EID_50_ /0.2 mL/bird	8 weeks
G4c	5 broiler chickens in contact with G4p (placed in contact with infected pigeons 24 h after being IN infected)	Contact infection	
G5p	10 pigeons left as non-infected pigeon controls	Nd	
G5c	10 broiler chickens left as non-infected chicken controls	Nd	

c: chickens, p: pigeons, IM: Intramuscularly infected, IN: Intranasally infected, nd: not done.

**Table 2:** The clinical and PM lesion sign scores of experimentally-infected birds.

Group	Severity of Clinical Signs			PM Lesions			
	Depression	Greenish Diarrhoea	Gasping	Nervous Signs	Tracheitis & Pneumonia (Hepatized Congested Lung)	Proventricular Haemorrhage	Button-like Ulcers in Intestine
G1c	+++	+++	+++	+++	+++	+++	+++
G2c	+++	+++	+++	+++	+++	+++	+++
G3p	+	+++	+	+++	++	-	-
G3c	++	++	+++	+++	+++	++	++
G4p	-	+++	+	+++	++	-	-
G4c	++	++	++	++	++	+ +	-
G5p	-	-	-	-	-	-	-
G5c	-	-	-	-	-	-	-

Respiratory signs were conjunctivitis, head swelling, gasping, sneezing, ocular and nasal discharge. Nervous signs were tremors, leg paralysis, torticollis, and circling. + + +, Severe; + +, Moderate; +, Mild; -, No signs or PM lesions.

## Abbreviations

IN: intranasally, IM: intramuscularly, NDV: Newcastle disease virus, NDV-VIId: Newcastle disease virus-genotype VIId, Dpi: Days post-infection, OIE: Office International des Epizooties, HA: haemagglutination, HI: haemagglutination inhibition, rRT-PCR: real-time reverse transcription polymerase chain reaction, G1c: group 1c: 10 broiler chickens infected intramuscularly (IM), G2c: group 2c: 10 broiler chickens infected intranasally (IN), G3p: group 3p: 15 pigeons infected IM in contact with 5 chickens, G3c: group 3c: 5 broiler chickens in contact with G3p, G4p: group 4p: 15 pigeons infected IN in contact with 5 chickens G4c, G4c: group 4c: 5 broiler chickens in contact with G4p, G5p: group 5p: 10 non-infected pigeons as controls., G5c: group 5c: 10 non-infected broiler chickens as controls, F: fusion, PM: post-mortem.
